# Supplementary material for: Malaria and helminth co-infections in children living in endemic countries: A systematic review with meta-analysis
Source: PLoS Negl Trop Dis. 2021 Feb 18;15(2):e0009138. doi: 10.1371/journal.pntd.0009138 (PMC7924789; doi:10.1371/journal.pntd.0009138)
Supplement: S1 Fig — (DOCX) [file pntd.0009138.s001.docx]

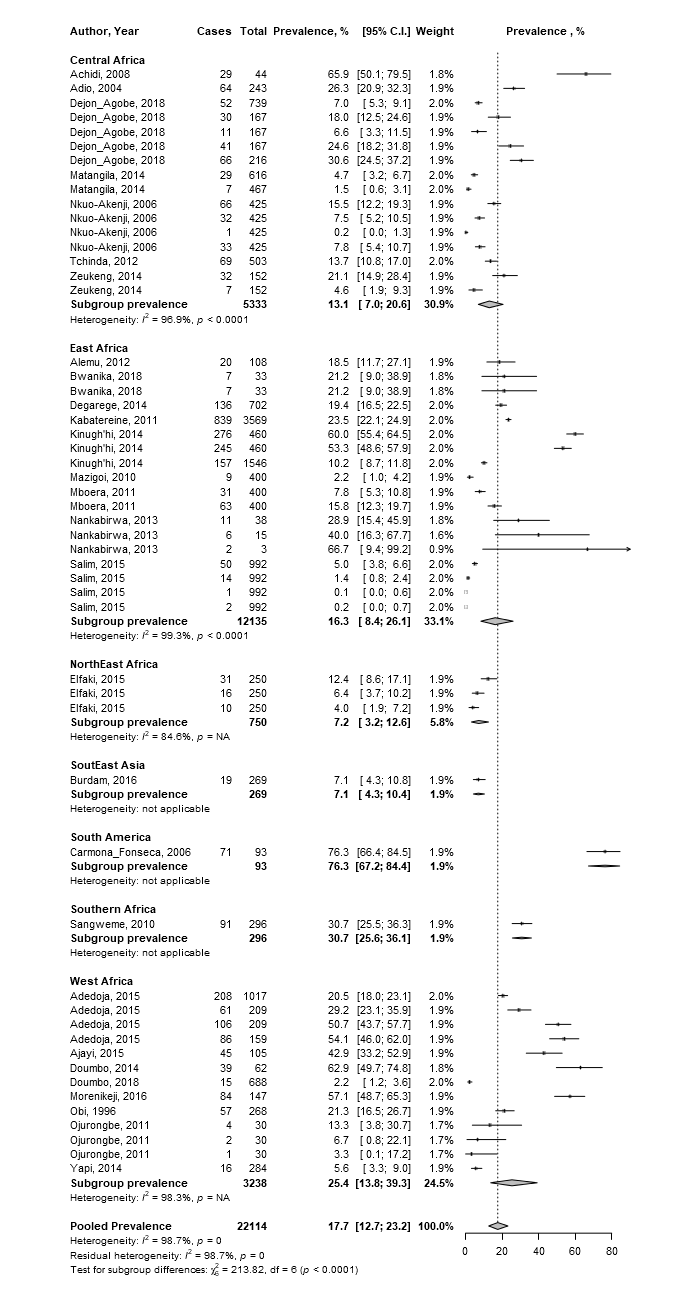


S1_Fig 1: Forest plot showing pooled prevalence of malaria-helminth co-infection by geographic region
