# Supplementary material for: Malaria and helminth co-infections in children living in endemic countries: A systematic review with meta-analysis
Source: PLoS Negl Trop Dis. 2021 Feb 18;15(2):e0009138. doi: 10.1371/journal.pntd.0009138 (PMC7924789; doi:10.1371/journal.pntd.0009138)

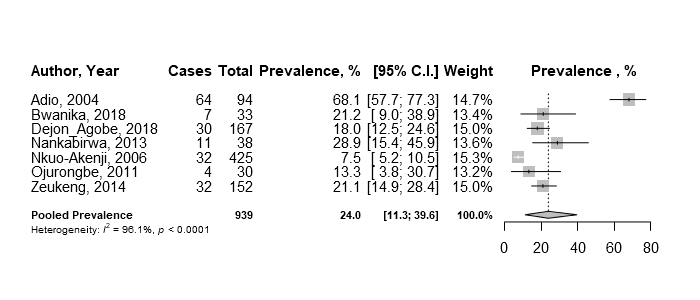


S2 Fig 2b: Forest plot showing sub-group analysis of summary estimates of prevalence of *P.falciparum*-hookworm co-infection

S2 Fig 2a: Forest plot showing sub-group analysis of summary estimates of prevalence of *P.falciparum-A. lumbricoides* co-infection


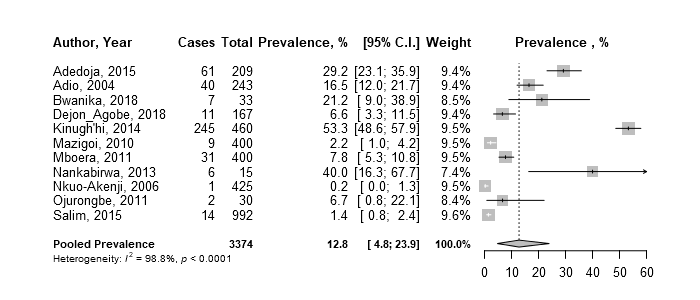


S2 Fig 2c: Forest plot showing sub-group analysis of summary estimates of prevalence of *P.falciparum-T.trichuria* co-infection


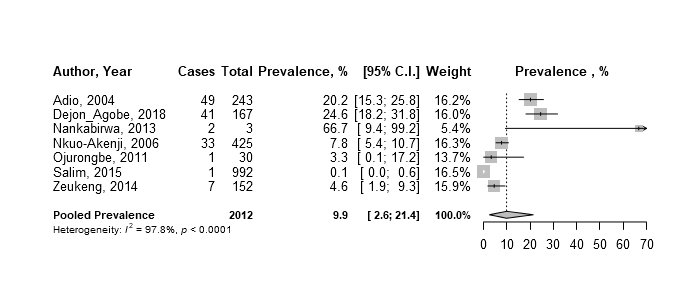

Supplement: S2 Fig — a-b-c: Forest plot showing sub-group analysis of summary estimates of prevalence of P.falciparum-A. lumbricoides co-infection, forest plot showing sub-group analysis of summary estimates of prevalence of P.falciparum-hookworm co-infection, forest plot showing sub-group analysis of summary estimates of prevalence of P.falciparum-T.trichuria co-infection. (DOCX) [file pntd.0009138.s002.docx]
