# Supplementary material for: Malaria and helminth co-infections in children living in endemic countries: A systematic review with meta-analysis
Source: PLoS Negl Trop Dis. 2021 Feb 18;15(2):e0009138. doi: 10.1371/journal.pntd.0009138 (PMC7924789; doi:10.1371/journal.pntd.0009138)

S3 Fig 3a: Forest plot showing sub-group analysis of prevalence of *Plasmodium-S. haematobium* co-infection in children in LMIC


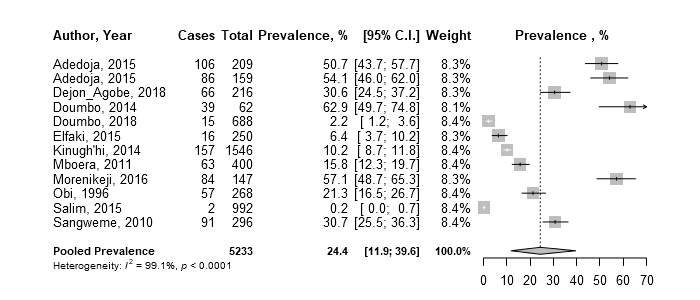


S3 Fig 3b: Forest plot showing sub-group analysis of prevalence of *Plasmodium-S.mansoni* co-infection in children in LMIC


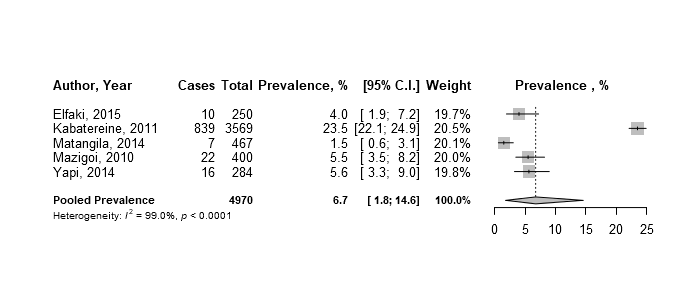

Supplement: S3 Fig — a-b: Forest plot showing sub-group analysis of prevalence of Plasmodium-S. haematobium co-infection in children in LMIC, forest plot showing sub-group analysis of prevalence of Plasmodium-S.mansoni co-infection in children in LMIC. (DOCX) [file pntd.0009138.s003.docx]
