# Supplementary material for: Malaria and helminth co-infections in children living in endemic countries: A systematic review with meta-analysis
Source: PLoS Negl Trop Dis. 2021 Feb 18;15(2):e0009138. doi: 10.1371/journal.pntd.0009138 (PMC7924789; doi:10.1371/journal.pntd.0009138)

S5 Fig: Forest plot showing the risk of asymptomatic/uncomplicated or severe *P. falciparum* infection in children co-infected with STH compared with children who did not have STH infection when adjusted for age, gender, socio-economic status and nutrition status of the children.


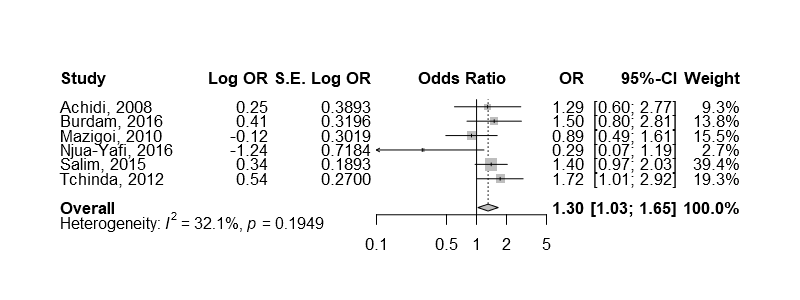

Supplement: S5 Fig — (DOCX) [file pntd.0009138.s005.docx]
