# Supplementary material for: Malaria and helminth co-infections in children living in endemic countries: A systematic review with meta-analysis
Source: PLoS Negl Trop Dis. 2021 Feb 18;15(2):e0009138. doi: 10.1371/journal.pntd.0009138 (PMC7924789; doi:10.1371/journal.pntd.0009138)
Supplement: S1 File — (PDF) [file pntd.0009138.s006.pdf]

IMPACT OF MALARIA-HELMINTH CO-INFECTIONS AMONG CHILDREN IN LOW AND MIDDLE INCOME COUNTRIES

A SYSTEMATIC REVIEW AND META-ANALYSIS

Study Protocol

Version 0.3; 20 Mar 2020

VERSION HISTORY

Version	Date	Summary of changes/revision
0.1	24 Jan 2020	First draft
0.2	03 Feb 2020	Addition of first independent reviewer<br>Revision of the search strategy with inclusion of ESPEN
0.3	20 Mar 2020	Addition of second independent reviewer

INVESTIGATORS

Name	Position and affiliation	Contact
Muhammed Afolabi	Assistant Professor, London School of Hygiene and Tropical Medicine, Faculty of Infectious and Tropical Diseases, Disease Control Department, Keppel Street, London WC1E 7HT	Tel: +44 207299 4834<br>E-mail: Muhammed.Afolabi@lshtm.ac.uk
Brian Greenwood	Professor of Clinical Tropical Medicine, London School of Hygiene and Tropical Medicine, Faculty of Infectious and Tropical Diseases, Disease Control Department, Keppel Street, London WC1E 7HT	Tel: +44 207299 4707<br>E-mail: Brian.Greenwood@lshtm.ac.uk
Amaya Bustinduy	Associate Professor, London School of Hygiene and Tropical Medicine, Faculty of Infectious and Tropical Diseases, Clinical Research Department, Keppel Street, London WC1E 7HT	Tel: +44 207927 2220<br>E-mail: Amaya.Bustinduy@lshtm.ac.uk
Rachel Pullan	Associate Professor, London School of Hygiene and Tropical Medicine, Faculty of Infectious and Tropical Diseases, Disease Control Department, Keppel Street, London WC1E 7HT	Tel: +44 207927 2702<br>E-mail: Rachel.Pullan@lshtm.ac.uk
Jane Falconer	User Support Services Librarian<br>Library & Archives Service<br>London School of Hygiene & Tropical Medicine<br>Keppel Street, London. WC1E 7HT	Tel: +44 207927 2414<br>E-mail: Jane.Falconer@lshtm.ac.uk
Edgard Dabira	Clinical Trial Coordinator<br>Disease Control and Elimination Theme<br>Medical Research Council, The Gambia @ LSHTM<br>Atlantic Boulevard, Fajara,<br>The Gambia	Tel: +220-2040284<br>E-mail: edabira@mrc.gm
Boni Maxime Ale	Medical Statistician & Epidemiologist<br>Holo Healthcare Ltd, Nairobi, Kenya	Tel : +254-707691490<br>E-mail : boniale@holo-healthcare.com

Table of Contents

1. INTRODUCTION 4
Background 4
1.2 Rationale for review 5
1.3 Aim and Objective 6
2. METHODS 6
2.1 Search Strategy 6
2.2 Search methods 6
2.3 Search Terms 7
2.4 Selection Criteria 9
2.5 Selection of studies 9
2.5 Data Extraction 10
2.7 Quality assessment/ risk of bias in individual studies 10
2.8 Data Synthesis 11
2.9 Registration and Reporting 11
2.10 Ethical considerations 11
2.11 Dissemination of findings 11
2.12 Timelines 12
References 13
Appendix 1: PRISMA checklist 15
Appendix 2: Newcastle-Ottawa Scale 17

1. INTRODUCTION

Background

In 2018, an estimated 228 million cases of malaria occurred globally with sub-Saharan Africa (SSA) and south-east Asia accounting for 93% and 3.4% respectively (1). *Plasmodium falciparum* remains the most prevalent malaria parasite in the WHO African Region, accounting for 99.7% of the estimated malaria cases in 2018, as well as in the WHO South-East Asia Region (50%). Globally, children aged under 5 years of age are the most vulnerable group affected by malaria. In 2018, they accounted for 67% (272 000) of all malaria deaths worldwide (1).

Adding to this very high burden is the co-existence of parasitic worms (helminths) with malaria among children in low and mid-income countries (2). Globally, an estimated 1.45 billion individuals are infected with these worm (3), with the most pervasive amongst these being soil-transmitted helminths (STH) which primarily comprise hookworm (*Ancylostoma duodenale* and *Necator americanus*), roundworm (*Ascaris lumbricoides*), and whipworm (*Trichuris trichiura*)(4). Other important helminths co-existing with malaria in children include *Schistosoma haematobium and S.mansoni* (5).

STH alone account for a global burden of over 3.3 million disability-adjusted life years (3) and are associated with anaemia, malnutrition, and impaired physical and cognitive development among preschool-aged children (PSAC), and school-aged children (SAC) (6-8). Furthermore, empirical evidence shows that STH have a negative impact on the clinical outcome of malaria, especially among PSAC and SAC (9) . Infections with Schistosomes and STH have also been reported to exert deleterious effects on the course and outcome of clinical malaria (10). A longitudinal study amongst Gabonese school children showed that *Trichuris trichiura* or hookworm associated with schistosomiasis increased the risk of *Plasmodium falciparum* infection; whilst schistosomiasis is independently associated with a malaria increase in young children (11).

Statistical and spatial models support the geographic overlap and co-endemicity of falciparum malaria and hookworm infections in SSA and suggest that about 25% of school-age children are at risk of these two groups of infection (12). Similar spatial distribution has been documented for the association between malaria and schistosomiasis (8). Hookworm infection and schistosomiasis are both known to cause anaemia (2). Similarly, malaria is also a leading cause of severe anaemia. Because the anaemia results from blood loss, haemolysis, an inflammatory process, and splenic sequestration, the additive low haemoglobin effect of the co-infections probably contributes substantially to mortality from malaria in children in SSA (13). Therefore, in low and mid income countries (LMIC) where malaria and helminths co-exist, there is potential for these infections acting

together to cause severe anaemia, a situation previously described as a 'perfect storm of anaemia' (14).

1.2 Rationale for review

Despite the obvious effects of helminths on malaria infection, the impact of these co-infections is not clearly elucidated as the few existing studies report conflicting findings on the association between malaria and helminths. Given the variability in the mechanisms of immune activation by helminths and *Plasmodium* parasites within human host (15), and the fact that helminths downregulate immune responses to *Plasmodium* pathogens, it can logically be concluded that STH infection increase risk to *Plasmodium* infection and related clinical outcomes. Nevertheless, the complex interactions between helminths and malaria during co-infections involving multiple pathways make the association and impact of the co-infections unclear (4, 5). While some studies reported protective effect of hookworms and *S. haematobium* infection (11, 16) against *Plasmodium* infection, others reported increased *Plasmodium* infection in children infected with *S. mansoni (10, 17)*.

In 2016, two systematic reviews with meta-analysis (4, 5) were published to address the limitations identified in the findings of two previous narrative reviews on the nature of the interactions and impact of malaria-helminth co-infections (18, 19). Most of the studies included in the systematic reviews published in 2016 were cross-sectional in nature, making it difficult to conclude whether the observed high prevalence and density of *P. falciparum* infections and low prevalence *P. falciparum*-related anaemia were due to STH infection. A moderate level of bias was also observed within the studies included in the reviews, thereby leading to an overestimation of the evidence of relationship reported between STH and asymptomatic/uncomplicated *P. falciparum* malaria in the reviews. Possible diagnostic inaccuracies for STH and malaria in the original studies included in the reviews might also have affected the conclusions, and the original studies may not have fully controlled the effect of different confounders that could affect the nature of relationship of STH and malaria (4, 5).

To overcome these limitations, this systematic review will make use of new and innovative methods to generate real-time epidemiologic profiles of malaria and helminth co-infections amongst children in low and mid-income countries. This approach has the potential to augment the traditional database search that is usually fraught with the limitations of original studies included in systematic reviews. This review will improve understanding on the effect of *P.falciparum* and helminth co-infections on the epidemiology of malaria among children living in LMIC. This would strengthen the evidence towards developing and implementing integrated malaria-helminth control strategies in high-burden settings.

1.3 Aim and Objective

The objective of this study is to systematically review available data from new mapping tools on malaria and helminths and the literature from cross-sectional studies, cohort studies and clinical trials on the nature of interactions between *P. falciparum* malaria and helminth infections (STH and *Schistosoma spp*) among children living in LMIC.

2. METHODS

2.1 Search Strategy

A systematic literature search will be conducted for scientific articles published in peer-reviewed journals, which reported *P. falciparum* malaria and helminth infections (STH and *Schistosoma* spp) among children living in LMIC. Conference abstracts without published full-texts will be excluded from the study. To allow for more eligible articles, no date and language restrictions will be applied on the search.

2.2 Search methods

Electronic databases: The following databases will be searched: Medline, EMBASE, Global Health and Web of Science for publications and conference abstracts using search terms described in Table 1. Further search will be conducted using innovative mapping databases including the Malaria Atlas Project (https://malariaatlas.org/); Global Atlas of Helminth Infections (https://www.ntd-ngonetwork.org/global-atlas-of-helminth-infections) and Expanded Special Project on Elimination of Neglected Tropical Diseases (ESPEN) (http://espen.afro.who.int/).

Other electronic sources: We will also review clinical trials' registries and platforms for malaria-helminth co-infections, including the WHO International Clinical Trials Registry Platform (ICTRP), the ClinicalTrials.gov registry, the Cochrane Central Register of Controlled Trials, systematic review registers (www.crd.york.ac.uk/PROSPERO) and meta-Register of controlled trials (ISRCTN registry). To track more relevant citations, grey literature and a list of references of potentially relevant papers will be searched and potentially relevant citations will be included for eligibility screening.

Hand searches: Reference lists of all full-text articles identified for inclusion in the systematic review will be screened, as well as reference lists of relevant review articles.

Compilation of results: Searches of all sources described above will be completed by MA; and submitted for peer review by LSHTM Library Services to confirm that no relevant papers have been left out. Details of each search will be fully documented (including, but not limited to, information

on search date, search strategy and number of results). Results (including titles and abstracts) will be compiled in Endnote X9, and any duplicates will be excluded.

2.3 Search Terms

A compound search strategy combining related truncated and non-truncated terms or synonyms will be used, and these will be tailored to each of the selected databases. For example, common terms (keywords or Medical Subject Headings-MeSH words) for malaria-helminth co-infection will be combined with the other key concepts identifying this condition in children living in LMIC, using the Boolean operator – "OR" or "AND" when appropriate to the specific database. The proposed search strategy is summarized in **Table 1**.

Table 1 – Proposed Search terms

Concept		Search terms including operators
Concept 1: **Malaria**	1.	malaria
	2.	Plasmodium
	3.	"Plasmodium falciparum"
	4.	Plasmodium vivax
	5.	malaria OR Plasmodium OR Plasmodium falciparum OR "Plasmodium vivax
Concept 2: **Helminths**	6.	soil-transmitted helminth
	7.	geohelminth
	8.	Ascaris
	9.	Ascaris lumbricoides
	10.	Trichuris
	11.	Trichuris trichiura
	12.	hookworm
	13.	Ancylostoma
	14.	Ancylostoma duodenale
	15	Necator
	16.	Necator americanus
	17.	Schistosomiasis
	18.	Schistosoma h*matobium
	19.	Schistosoma masoni
	20.	Bilharziasis
	21.	soil-transmitted helminth OR geohelminth OR Ascaris OR Ascaris lumbricoides OR Trichuris OR Trichuris trichiura OR hookworm OR Ancylostoma OR Ancylostoma duodenale OR Necator OR Necator americanus OR Schistosomiasis OR Schistosoma h*matobium OR Schistosoma masoni OR Bilharziasis
Concept 3: **Children**	22.	Children
	23.	School-aged children
	24.	Pre-school aged children
	25.	Under-five children
	26.	Paediatric
	27.	Pediatric
	28.	Children OR School-aged children OR Pre-school aged children OR Under-five children OR Paediatric OR Pediatric
Concept 4: **Low and mid-income countries**	29.	Expert filters built by LSHTM Library services will be used. All countries designated as LMIC (https://ovidsp.dc1.ovid.com/sp-4.04.0a/ovidweb.cgi)
**Combined terms**	30.	5 AND 21 AND 28 AND 29

The complete citations of all search results will be exported to EndNote X9 (Thomson Reuters, New York, USA) reference manager, after which duplicates will be removed.

2.4 Selection Criteria

The PICOST framework will be used to aid the selection of published articles relevant to the search question eligible for the review:

Population of interest: pre-school and school aged children

Intervention (Exposure): malaria and helminth co-infections

Comparator: None

Outcome: anaemia, poor physical and cognitive development, death

Setting: low and mid-income countries

Type of study: Observational studies, interventional studies, systematic reviews, Cochrane reports

Inclusion criteria

1. Articles which are published in peer-reviewed journals.
2. Articles which described primary data findings from interventional or observational studies.

Exclusion criteria.

1. Case reports and case series
2. Studies whose full-texts could not be retrieved.
3. Duplicate articles describing the same subjects.

2.5 Selection of studies

The literature search will be conducted as described above by MA. All articles returned by the database search will be screened independently for eligibility by two reviewers (ED, BMA) based on titles and abstracts using the checklist summarized in Table 2, to exclude obviously non-relevant articles. There will be no restriction on study designs. Following this step, full-texts of the studies focusing on malaria-helminth co-infections will be accessed through the LSHTM Library and Archives and assessed for eligibility using the above-listed criteria.

Table 2. Checklist for elimination of study by abstract screening.

	Questions	Yes	No	Unclear
1.	Is the publication in a peer-reviewed journal?			
2.	Was the article describing malaria and helminth co-infections?			
3.	If the study was on malaria-helminth co-infections, did it also focus on the impact of the co-infections?			
4.	Did the study report the risk, clinical course or interactions between malaria and helminth infections			
5.	Did the study involve paediatric population as defined above?			
	*If the answer to any of the questions 1-5 above is 'No' then the study is not eligible.<br><br>*If 'No' to all, but one 'Unclear', then retrieve the full-text article for clarity.			

2.5 Data Extraction

Data will be extracted using a standard form. To ensure there are no errors during the extraction process, the extracted data will be re-checked for accuracy by MA. Irrespective of the study design, the following data will be extracted from all included studies: first author and year of publication, country/setting, study title and objectives; confirmation of eligibility for review; methodology - study design, sample size, study duration, data collection with study time points; population of interest; interventions; outcome - indicators; results; authors key discussions /comments / limitations; reviewer's comments.

2.7 Quality assessment/ risk of bias in individual studies

To ascertain the internal and external validity of the eligible studies, including the risk of bias, the quality of each study will be assessed using a combination of Newcastle Ottawa Scale (NOS) (20) and Effective Public Health Practice Project (EPHHP) tools (21), which are recommended for systematic reviews by The Cochrane Public Health Review Group (22). The NOS has been adapted for use in cross-sectional studies from the Newcastle-Ottawa Quality Assessment Scale for cohort studies to perform a quality assessment of cross-sectional studies for systematic review in other studies. Therefore, it will be used to assess the cross-sectional studies in this review. Studies will be assessed from three angles: selection of the study groups, comparability of the study groups, and the ascertainment of the outcome of interest. The studies will be awarded a score – weak, moderate, or strong, for the overall quality. Each study will be assessed for the following: selection bias, study

design, confounding, blinding, data collection methods, and withdrawals and drop-outs. The scale is summarized in Appendix 2.

2.8 Data Synthesis

Narrative synthesis: Narrative synthesis will be carried out alongside meta-analysis, using a framework which consists of three elements: 1. developing a preliminary synthesis of findings of included studies; 2. exploring relationships within and between studies; and 3. assessing the robustness of the synthesis. Studies will be grouped together if they compared similar types of outcomes of interest. Where available, references to p-values and confidence intervals of observed associations will be made in relation to their strengths and limitations.

Meta-analysis: Where feasible, considering potential heterogeneity in study designs, we will perform meta-analyses for the outcomes of malaria and helminth co-infections and nature of interactions between malaria and helminths. We will use random-effects meta-analysis to estimate pooled effect estimates across studies, allowing for between-study heterogeneity (23). We will examine heterogeneity using the I^2 statistic and publication bias using funnel plots and Begg's test for correlation between the effect estimate and their variances (24).

An influence analysis will also be performed to assess the robustness of the pooled summary effects by excluding each of the studies from the pooled estimate. Primary analyses will be stratified by gender. Sub-group analyses by (i) age and (ii) species of helminths will be performed to compare pooled effects and heterogeneity. Data will be analyzed using Stata version 16.0 (Stata Statistical Software, College Station. TX: Stata Corporation).

2.9 Registration and Reporting

The protocol for this systematic review will be registered on PROSPERO (https://www.crd.york.ac.uk/PROSPERO/). The review will be reported according to the Preferred Reporting Items for Systematic Reviews and Meta-Analyses (PRISMA) (25).

2.10 Ethical considerations

As this is a systematic review of published articles, ethical approval is not required.

2.11 Dissemination of findings

Findings of the data extracted from eligible studies in this systematic review will be disseminated at scientific meetings and in a peer-reviewed journal.

2.12 Timelines

Timelines for completion of the work described in this protocol are shown in Table 3

	Month 1	Month 2	Month 3	Month 4	Month 5	Month 6	Month 7
Finalise and register protocol on PROSPERO							
Perform database search							
Screen titles and abstracts							
Review full text publications							
Data extraction and analysis							
Manuscript write-up							

Table 3: Gantt chart showing estimated timelines for conducting the systematic review activities.

Timelines may vary according to numbers of search hits and full papers to be reviewed.

References

1. World malaria report 2019. Geneva: World Health Organization; 2019. Licence: CC BY-NC-SA 3.0 IGO. Available online at https://www.who.int/publications-detail/world-malaria-report-2019. Accessed 20 Jan 2020.
2. Brooker S, Akhwale W, Pullan R, Estambale B, Clarke SE, Snow RW, et al. Epidemiology of Plasmodium-Helminth Co-Infection in Africa: Populations at Risk, Potential Impact on Anemia, and Prospects for Combining Control. The American Journal of Tropical Medicine and Hygiene. 2007;77(6_Suppl):88-98.
3. 2030 targets for soil-transmitted helminthiases control programmes. Geneva: World Health Organization; 2019. Licence: CC BY-NC-SA 3.0 IGO. Available online at https://www.who.int/intestinal_worms/resources/9789240000315/en/. Accessed 20 Jan 2020.
4. Degarege A, Veledar E, Degarege D, Erko B, Nacher M, Madhivanan P. Plasmodium falciparum and soil-transmitted helminth co-infections among children in sub-Saharan Africa: a systematic review and meta-analysis. Parasit Vectors. 2016;9(1):344.
5. Degarege A, Degarege D, Veledar E, Erko B, Nacher M, Beck-Sague CM, et al. Plasmodium falciparum Infection Status among Children with Schistosoma in Sub-Saharan Africa: A Systematic Review and Meta-analysis. PLoS neglected tropical diseases. 2016;10(12):e0005193.
6. Degarege A, Animut A, Legesse M, Medhin G, Erko B. Malaria and helminth co-infection and nutritional status of febrile patients in Southern Ethiopia. Journal of infection and public health. 2014;7(1):32-7.
7. Degarege A, Legesse M, Medhin G, Animut A, Erko B. Malaria and related outcomes in patients with intestinal helminths: a cross-sectional study. BMC infectious diseases. 2012;12:291.
8. Kabatereine NB, Standley CJ, Sousa-Figueiredo JC, Fleming FM, Stothard JR, Talisuna A, et al. Integrated prevalence mapping of schistosomiasis, soil-transmitted helminthiasis and malaria in lakeside and island communities in Lake Victoria, Uganda. Parasites & Vectors. 2011;4(1):232.
9. Hürlimann E, Yapi RB, Houngbedji CA, Schmidlin T, Kouadio BA, Silué KD, et al. The epidemiology of polyparasitism and implications for morbidity in two rural communities of Côte d'Ivoire. Parasites & Vectors. 2014;7(1):81.
10. Sangweme DT, Midzi N, Zinyowera-Mutapuri S, Mduluza T, Diener-West M, Kumar N. Impact of schistosome infection on Plasmodium falciparum Malariometric indices and immune correlates in school age children in Burma Valley, Zimbabwe. PLoS neglected tropical diseases. 2010;4(11):e882.
11. Dejon-Agobe JC, Zinsou JF, Honkpehedji YJ, Ateba-Ngoa U, Edoa JR, Adegbite BR, et al. Schistosoma haematobium effects on Plasmodium falciparum infection modified by soil-transmitted helminths in school-age children living in rural areas of Gabon. PLoS neglected tropical diseases. 2018;12(8):e0006663.
12. Degarege A, Erko B. Epidemiology of Plasmodium and Helminth Coinfection and Possible Reasons for Heterogeneity. BioMed research international. 2016;2016:3083568.
13. Ezeamama AE, McGarvey ST, Acosta LP, Zierler S, Manalo DL, Wu HW, et al. The synergistic effect of concomitant schistosomiasis, hookworm, and trichuris infections on children's anemia burden. PLoS neglected tropical diseases. 2008;2(6):e245.
14. Hotez PJ, Molyneux DH. Tropical anemia: one of Africa's great killers and a rationale for linking malaria and neglected tropical disease control to achieve a common goal. PLoS neglected tropical diseases. 2008;2(7):e270.
15. Hartgers FC, Yazdanbakhsh M. Co-infection of helminths and malaria: modulation of the immune responses to malaria. Parasite immunology. 2006;28(10):497-506.
16. Lyke KE, Dicko A, Dabo A, Sangare L, Kone A, Coulibaly D, et al. Association of Schistosoma haematobium infection with protection against acute Plasmodium falciparum malaria in Malian children. Am J Trop Med Hyg. 2005;73(6):1124-30.
17. Sokhna C, Le Hesran JY, Mbaye PA, Akiana J, Camara P, Diop M, et al. Increase of malaria attacks among children presenting concomitant infection by Schistosoma mansoni in Senegal. Malaria journal. 2004;3:43.
18. Adegnika AA, Kremsner PG. Epidemiology of malaria and helminth interaction: a review from 2001 to 2011. Current opinion in HIV and AIDS. 2012;7(3):221-4.
19. Nacher M. Interactions between worms and malaria: good worms or bad worms? Malaria journal. 2011;10:259.
20. Wells GA, Shea B, O'Connell D, Peterson J, Welch V, Losos M, et al. The Newcastle-Ottawa Scale (NOS) for assessing the quality of nonrandomised studies in meta-analyses 2014 [updated 2014; cited 2018 01/12/2018]. Available from: http://www.ohri.ca/programs/clinical_epidemiology/oxford.asp.
21. Effective Public Health Practice Project. Effective Public Health Practice Project. (1998). Dictionary for the Effective Public Health Practice Project Quality Assessment Tool For Quantitative Studies, [Accessed from

http://www.ephpp.ca/PDF/QADictionary_dec2009.pdf], [Date Accessed: 20/08/2015] 1998 [cited 2015 20/08/2015]. Available from: http://www.ephpp.ca/PDF/QADictionary_dec2009.pdf.
22. Armstrong R, eds. Ch 21: . Available from: www.cochrane-handbook.org. Reviews in health promotion and public health. Cochrane Handbook for Systematic Reviews of Interventions. [Accessed from www.cochrane-handbook.org] [Date Accessed: 20/08/2015]: The Cochrane Collaboration; 2008 [cited 2015 20/08/2015]. Available from: www.cochrane-handbook.org.
23. Egger M, Smith GD, Phillips AN. Meta-analysis: principles and procedures. BMJ (Clinical research ed). 1997;315(7121):1533-7.
24. Begg CB, Mazumdar M. Operating characteristics of a rank correlation test for publication bias. Biometrics. 1994;50(4):1088-101.
25. Moher D, Liberati A, Tetzlaff J, Altman DG, Group P. Preferred reporting items for systematic reviews and meta-analyses: the PRISMA statement. PLoS Med. 2009 Jul 21;6(7):e1000097.

Appendix 1: PRISMA checklist

Section/topic	#	Checklist item	Reported on page #
**TITLE**			
Title	1	Identify the report as a systematic review, meta-analysis, or both.	
**ABSTRACT**			
Structured summary	2	Provide a structured summary including, as applicable: background; objectives; data sources; study eligibility criteria, participants, and interventions; study appraisal and synthesis methods; results; limitations; conclusions and implications of key findings; systematic review registration number.	
**INTRODUCTION**			
Rationale	3	Describe the rationale for the review in the context of what is already known.	
Objectives	4	Provide an explicit statement of questions being addressed with reference to participants, interventions, comparisons, outcomes, and study design (PICOS).	
**METHODS**			
Protocol and registration	5	Indicate if a review protocol exists, if and where it can be accessed (e.g., Web address), and, if available, provide registration information including registration number.	
Eligibility criteria	6	Specify study characteristics (e.g., PICOS, length of follow-up) and report characteristics (e.g., years considered, language, publication status) used as criteria for eligibility, giving rationale.	
Information sources	7	Describe all information sources (e.g., databases with dates of coverage, contact with study authors to identify additional studies) in the search and date last searched.	
Search	8	Present full electronic search strategy for at least one database, including any limits used, such that it could be repeated.	
Study selection	9	State the process for selecting studies (i.e., screening, eligibility, included in systematic review, and, if applicable, included in the meta-analysis).	
Data collection process	10	Describe method of data extraction from reports (e.g., piloted forms, independently, in duplicate) and any processes for obtaining and confirming data from investigators.	
Data items	11	List and define all variables for which data were sought (e.g., PICOS, funding sources) and any assumptions and simplifications made.	
Risk of bias in individual studies	12	Describe methods used for assessing risk of bias of individual studies (including specification of whether this was done at the study or outcome level), and how this information is to be used in any data synthesis.	
Summary measures	13	State the principal summary measures (e.g., risk ratio, difference in means).	
Synthesis of results	14	Describe the methods of handling data and combining results of studies, if done, including measures of consistency (e.g., I^2) for each meta-analysis.	

Section/topic	#	Checklist item	Reported on page #
Risk of bias across studies	15	Specify any assessment of risk of bias that may affect the cumulative evidence (e.g., publication bias, selective reporting within studies).	
Additional analyses	16	Describe methods of additional analyses (e.g., sensitivity or subgroup analyses, meta-regression), if done, indicating which were pre-specified.	
**RESULTS**			
Study selection	17	Give numbers of studies screened, assessed for eligibility, and included in the review, with reasons for exclusions at each stage, ideally with a flow diagram.	
Study characteristics	18	For each study, present characteristics for which data were extracted (e.g., study size, PICOS, follow-up period) and provide the citations.	
Risk of bias within studies	19	Present data on risk of bias of each study and, if available, any outcome level assessment (see item 12).	
Results of individual studies	20	For all outcomes considered (benefits or harms), present, for each study: (a) simple summary data for each intervention group (b) effect estimates and confidence intervals, ideally with a forest plot.	
Synthesis of results	21	Present results of each meta-analysis done, including confidence intervals and measures of consistency.	
Risk of bias across studies	22	Present results of any assessment of risk of bias across studies (see Item 15).	
Additional analysis	23	Give results of additional analyses, if done (e.g., sensitivity or subgroup analyses, meta-regression [see Item 16]).	
**DISCUSSION**			
Summary of evidence	24	Summarize the main findings including the strength of evidence for each main outcome; consider their relevance to key groups (e.g., healthcare providers, users, and policy makers).	
Limitations	25	Discuss limitations at study and outcome level (e.g., risk of bias), and at review-level (e.g., incomplete retrieval of identified research, reporting bias).	
Conclusions	26	Provide a general interpretation of the results in the context of other evidence, and implications for future research.	
**FUNDING**			
Funding	27	Describe sources of funding for the systematic review and other support (e.g., supply of data); role of funders for the systematic review.	

Appendix 2: Newcastle-Ottawa Scale

Selection: (Maximum 5 stars)

1) Representativeness of the sample:

a) Truly representative of the average in the target population. * (all subjects or random sampling)

b) Somewhat representative of the average in the target population. * (non-random sampling)

c) Selected group of users.

d) No description of the sampling strategy.

2) Sample size:

a) Justified and satisfactory. *

b) Not justified.

3) Non-respondents:

a) Comparability between respondents and non-respondents characteristics is established, and the response rate is satisfactory. *

b) The response rate is unsatisfactory, or the comparability between respondents and non-respondents is unsatisfactory.

c) No description of the response rate or the characteristics of the responders and the non-responders.

4) Ascertainment of the exposure (risk factor):

a) Validated measurement tool. **

b) Non-validated measurement tool, but the tool is available or described.*

c) No description of the measurement tool.

Comparability: (Maximum 2 stars)

1) The subjects in different outcome groups are comparable, based on the study design or analysis. Confounding factors are controlled.

a) The study controls for the most important factor (select one). *

b) The study control for any additional factor. *

Outcome: (Maximum 3 stars)

1) Assessment of the outcome:

a) Independent blind assessment. **

b) Record linkage. **

c) Self report. *

d) No description.

2) Statistical test:

a) The statistical test used to analyze the data is clearly described and appropriate, and the measurement of the association is presented, including confidence intervals and the probability level (p value). *

b) The statistical test is not appropriate, not described or incomplete.
