# Supplementary material for: Malaria and helminth co-infections in children living in endemic countries: A systematic review with meta-analysis
Source: PLoS Negl Trop Dis. 2021 Feb 18;15(2):e0009138. doi: 10.1371/journal.pntd.0009138 (PMC7924789; doi:10.1371/journal.pntd.0009138)
Supplement: Attachment — Submitted filename: Response to reviewers.doc [file pntd.0009138.s007.doc]

11 December 2020

Dear Editor,

Revision of manuscript PNTD-D-20-01548

Malaria and helminth co-infections in children living in endemic countries: a systematic review with meta-analysis

Thank you for sharing with us the reviewers’ comments on the above named manuscript. We are pleased that the reviewers feel the manuscript is an interesting paper on an important topic, and we have carefully taken the editorial and reviewers’ comments and suggestions into account in the revision of the manuscript. We describe below how we have responded to specific comments.

We have addressed the concerns from the reviewers and editors, and we hope this paper is now acceptable for publication in your journal.

Yours sincerely,

Muhammed Afolabi

On behalf of all co-authors

**Reviewers' comments:**

**Reviewer #1:**

1. The objectives of the study are clearly articulated. The study design is appropriate to address the stated objectives. The population is clearly described and appropriate for the hypothesis. The sample size has added to the body of knowledge by further studies are recommended. Correct statistical analysis were used to support conclusions. Are there no concerns about ethical or regulatory requirements?

***Response****: We thank the reviewer for this positive feedback. Given that this study is a systematic review of published articles, ethical or regulatory approval is not required for the conduct of the systematic review, hence, we do not have any concern about ethical or regulatory requirement.*

**Reviewer #2**:

Abstract

2. Background: Line # 159-162: The authors need to describe the study objective clearly. It is a mixed of objective and utility of this review.

***Response****: We have described the objective more clearly. In lines 160-164 (of the clean copy of the revised manuscript), we have added the sentence: ‘The objective of this study was to systematically review available data on the burden of malaria-helminth co-infections and the nature of interactions between Plasmodium malaria and helminth infections (STH and Schistosoma spp) among children living in endemic countries, with a view to establishing how control of malaria and helminth infections might be better co-ordinated in the future.*

Methodology/Principal Findings

3. Line # 44-47 Information provided is not yet clear enough

***Response****: We have revised the information in lines 44-47 to make it clearer as follows: We used the summary odds ratio (OR) and 95% confidence intervals (CI) as a measure of association (random-effects model). We also performed Chi-square heterogeneity test based on Cochrane’s Q and evaluated the severity of heterogeneity using I^2^ statistics. The included studies were examined for publication bias using a funnel plot and statistical significance was assessed using Egger’s test (bias if p<0.1).*

4. Please, rephrase these sentences. The authors chose a random model due to a substantial heterogeneity among studies (i.e. I^2^ test more than?? %). Please, provide a relevant ref (e.g. Higgins et al 2019. Cochrane Handbook for Systematic Reviews of Interventions version 6)

***Response****: Higgins et al (2003) have suggested a rule-of-thumb to interpret I^2^ (I^2^ of 25% may represent low heterogeneity, 50% medium heterogeneity and 75% high heterogeneity). They also pointed out that a fixed rule-of-thumb of the interpretation of I^2^ may not be appropriate for all circumstances. There is no consensus that we are aware of about the thresholds of I^2^.*

*In the 2019 Cochrane Handbook in section 10.10.2 (Identifying and measuring heterogeneity), it is stated: “a rough guide to interpretation in* ***the context of meta-analyses of randomized trials*** *is as follows: 0% to 40%: might not be important; 30% to 60%: may represent moderate heterogeneity; 50% to 90%: may represent substantial heterogeneity; 75% to 100%: considerable heterogeneity”. We understood that these suggested thresholds might be applicable when performing a* ***meta-analysis of randomized trials****, which is not the case in our study.*

*What is known is that the higher the value, the greater the inconsistency across studies. Therefore, we did not interpret I^2^ based on pre-set thresholds.*

5. To be more informative, please provide I^2^ values (after OR and its 95%I) for important outcomes.

***Response****: We have provided* *I^2^ values (after OR and its 95%I) for important outcomes in the revised manuscript.*

6. Line # 58……………..It will be better to describe “A subset of 16 studies …..”

***Response****: As advised, we have described the sentence as follows: A subset of 16 studies showed that the odds of anaemia were higher………*

Text: Eligibility Criteria: Line # 172-76

7. Because of observational studies, PECO format should be more applicable. It will be better to present the inclusion criteria addressing each acronym For example, Participants ( P): children aged 1-16 years living in LMIC,

***Response****: We have revised the sentence in line with the reviewer’s comment as follows: The PECO framework [25] was used to aid the selection of published articles relevant to the search terms which were eligible for the review, where ‘P’ stands for the population (children aged 1-16 years living in endemic countries); ‘E’ for exposure (malaria and helminth co-infection); ‘C ‘ for comparator which is not applicable in the context of this review; and ‘O’ stands for outcome of interest (anaemia).*

8. Line # 177: Adults or pregnant women were excluded. To be more informative, please give justifications in the light of immune status or pathophysiology.

***Response****: We have provided the justifications for excluding adults or pregnant women. In lines 183-185, we added ‘Also excluded were studies which enrolled only adults or pregnant women, because pathophysiology and immunological effects of malaria and helminth infections differ significantly in children from adults and pregnant women [26].*

9. Line # 204-205: It will be better to provide kappa statistic.

***Response****: We did not perform Kappa statistics analysis to determine the degree of inter-observer agreement between the two colleagues who independently reviewed the articles for eligibility for inclusion in this review. We have therefore revised the sentence to read as follows: Disagreements between the two reviewers on the inclusion of a study for full text review were resolved following discussions with a third independent reviewer.*

Data Analysis

10. Line # 230: If adjusted OR was not available in an included study, how would you treat such study?

***Response****: In general, most of the included studies did not report adjusted OR. For a study in which adjusted OR was not available, we estimated the summary Mantel-Haenszel odds ratio of asymptomatic/uncomplicated or severe Plasmodium infection in children infected with STH or Schistosomes compared to those with Plasmodium infection only, to obtain the adjusted odds ratio.*

11. The authors did not provide how they had estimated a pooled prevalence. (On line # 293, the authors provided the results of pooled prevalence. If so, the method of pooling prevalence should have been described in the method section). For more details, please, see a ref (Nyaga VN, Arbyn M, Aerts M. Metaprop: a Stata command to perform meta-analysis of binomial data. Arch Public Health. 2014;72(1):39). This paper addressed tests of significance on the pooled proportion which typically rely on normal probabilities. It also, address Freeman-Tukey double arcsine transformation (this ref paper is aimed to address metaprop package in STATA. But, it is applicable for methodological aspect of pooled prevalence, regardless of software used.)

***Response****: Under the section on data analysis, we explained how we estimated a pooled prevalence. In lines 242-252, we explained in detail that: We extracted raw data for only malaria positive, only STH-positive, only S. haematobium or S. mansoni-positive, malaria-S. haematobium or S. mansoni-positive, malaria-and STH-positive, and intestinal helminth- and malaria-negative and used these data to estimate the pooled prevalence of malaria in the group of children who were malaria positive and STH/Schistosoma positive compared to the prevalence in those who were malaria positive and STH/Schistosoma negative. We also used the data obtained to calculate the crude odds of asymptomatic/uncomplicated or severe Plasmodium infection and related anaemia in children infected with Schistosomes or STH compared to those infected with Plasmodium parasites only, along with their 95% CI. The log OR and the standard error (SE) of the log OR were estimated using generic inverse variance weighting method [33] and the summary estimate (summary-odds ratio) was estimated’.*

*Moreover, we did not use Freeman-Tukey double arscine transformation because of potential problems in the back-transformation of meta-analysis results (Schwarzer G, Chemaitelly H, Abu-Raddad LJ, Rücker G. Seriously misleading results using inverse of Freeman-Tukey double arcsine transformation in meta-analysis of single proportions. Res Synth Methods. 2019 Sep;10(3):476-483. doi: 10.1002/jrsm.1348. Epub 2019 Apr 23. PMID: 30945438; PMCID: PMC6767151.).*

**Reviewer #3**:

12. The objectives of the study are clearly articulated with a clear testable hypothesis. The study design is appropriate to address the research question

***Response****: We note with thanks this positive feedback from the reviewer*

13. The population description is not appropriate for the hypothesis being tested, since the pathogens study or reviewed are not limited to the LMIC, rather to endemic regions. The age groups are not well define and seems to be limited to infants without explaining the rational to exclude adults or not.

***Response****: We have revised the description of the target population to make it clearer and provided justification for exclusion of adults. In lines 183-185, we added: Also excluded were studies which enrolled only adults or pregnant women, because pathophysiology and immunological effects of malaria and helminth infections differ significantly in children from adults and pregnant women [26].*

14. The title of the review including LMIC, but there is no rationale under this specification. Would co-infection of helminth and malaria differ in non-LMIC? One could simply consider in endemic areas.

***Response****: We thank the reviewer for their comment and agree with the suggestion about the title of this article. We have revised the title to read as follows: Malaria and helminth co-infections in children living in* ***endemic countries****: a systematic review with meta-analysis*

15. The keys word includes paediatrics population, which is too specific. Better consider pre- and school children

***Response****: We note this comment from reviewer with thanks.*

16. The sample size calculation is not applicable in the review. The statistical analysis is appropriate to support the conclusion. The ethical issue is not applicable. However the review is registered.

***Response****: We thank the reviewer and agree with their submission on the above three items.*

**Reviewer #1:**

17. The analysis presented match the analysis plan and the results are clearly and completely presented. However the data in Figure 2 presents P-Schisto first and P-STH afterwards which in the reverse to the data presented in the Abstract and the main text. I recommend reversing the data present in Figure 2 to match the flow of the text.

***Response****: In line with the reviewer’s recommendation, we have revised the data in Figure 2 to match the flow of the text in the abstract. In lines 52-56, we added: Summary estimates from 14 studies showed a lower odds of P. falciparum infection in children co-infected with Schistosoma spp (OR: 0.65; 95%CI: 0.37-1.16). Similar lower odds of P. falciparum infection were observed from the summary estimates of 24 studies in children co-infected with soil transmitted helminths (STH) (OR: 0.42; 95%CI: 0.28-0.64).*

*In lines 312-315, we added: Disaggregated by helminth types, the pooled prevalence of Plasmodium-Schistosoma co-infections in 9,803 children was 19.2% (95% CI: 9.6-31.1%); and the pooled prevalence of Plasmodium-STH co-infections in 12311 children was 17 % (95%CI: 11.4-23.4%); (95% CI: 9.6-31.1%) (Fig 2).*

18. The age range(s) in Table 1 for manuscripts 9, 10, 11, 23 and 44 are unclear.

***Response****: We have revised the age ranges in Table 1 for manuscripts 9, 10, 11, 23 and 44 to make them clearer*. Age range for manuscript 9 is 12- 59 months, for manuscript 10, it is 4-16 years, for 11, 4-16 years, for 23, 6 months – 9 years and for 44, 6 months – 15 years

**Reviewer #2:**

19. The current presentation is not yet smooth. It will be better to improve the flow of presentation. Having mentioned above, it will be better to provide I^2^ values.

***Response****: As highlighted in the responses above, we have provided the I^2^ values and revised the manuscript in line with the reviewers’ comments to improve the flow of the presentation.*

**Reviewer #3:**

20. The analysis presented match the analysis plan. In general the results are clearly presented, however, two sub title overlap. Line 334: Anaemia in *Plasmodium*- helminth co-infections" and line 365: Anaemia induced by malaria-helminth co-infection" . More distinction are needed.

***Response****: The two sub-titles belong to different sections of the manuscript. While line 334: ‘Anaemia in Plasmodium- helminth co-infections’ discussed the findings of the meta-analysis component; line 365: ‘Anaemia induced by malaria-helminth co-infection’ is the summary of the narrative reviews of articles in which meta-analysis could not be performed. To ensure greater clarity, we have revised the sub-titles to highlight the distinction. In line 346 of the revised manuscript, we revised the subtitle to read as follows: Risk of Anaemia in malaria-helminth co-infection.*

21. The other reviews on co-infection should be reviewed and taking in consideration to avoid the redundant since some facts are known already.

***Response****: We have considered carefully other existing systematic reviews on malaria-helminth co-infections and avoided unnecessary duplication of facts that are already known about the subject.*

**Reviewer #1**:

22. The conclusions are supported by the data presented. The limitations of the analysis (suitable published manuscripts from LMIC) are clearly described. The authors discuss how these data can be helpful to advance our understanding of the topic under study and the need for further studies and analysis.

***Response****: We thank the reviewers for these positive comments*

23. The public health relevance of immune-regulation should be further addressed and potentially its long-term implications if programs continue to make progress towards control/elimination.

***Response****: As requested, we have further addressed the public health relevance of immune regulation and long term complication of malaria-helminth co-infection. We added to the last sentence in the discussion…..Understanding the dynamics of immune regulation in malaria and helminth co-infection may also be useful in malaria vaccine development to* *understand* *the influence of the dual infection on vaccine immunogenicity (Brooker et al, 2007).*

**Reviewer #2:**

24. It is difficult to catch the salient points. It will be better to present with the use of subheadings, including study limitations.

***Response:*** *As requested, we have presented the discussion under subheadings*

**Reviewer #3:**

25. The conclusion are clear and supported by data presented. The limitation are described, as well as public health relevance.

***Response****: We thank the reviewer for their comments*

**Editorial and Data Presentation Modifications**

**Reviewer #1:**

26. Minor Revision: To address suggestions to Figure 2 and Table 1 and expand the discussion on immune regulation.

***Response****: We have addressed the reviewer’s comments on Figure 2 and Table 1, as highlighted above.*

**Summary and General Comments**

**Reviewer #1**: This is a well-researched and well-presented paper.

27. The topic is highly significant as the global burden of STH and Schito reduce and the complicated interaction with malaria, anaemia and possibly other infections such as Covid-19 are so little understood.

***Response:*** *We thank the reviewer for their comments*

**Reviewer #2**: It is fine.

**Reviewer #3**:

28. The review entitled " Malaria and helminth co-infections in children living in low- and middle-income countries: a systematic review with meta-analysis "undertaken by Dr. Afolabi et al, is useful and mark a new step regarding the rationale research question of co-infection of malaria and helminths in term of susceptibility and immune responses. However, some points need to be addressed ahead. The title of the review including LMIC, but there is no rationale under this specification. Would co-infection of helminth and malaria differ non-LMIC? One could simply consider in endemic areas

***Response****: We have revised the title in line with the suggestion of the reviewer*

29. The keys word includes paediatrics population, which is too specific. Better consider pre- and school children

***Response****: We note the reviewer’s comment with thanks*

30. On line 185, the egg detection methods are too limited. Please elaborate a bit more

***Response****: We have elaborated on the egg detection method. In lines 193-196, we have added*: *Kato-Katz thick smears were produced from the stool samples collected from the children and these samples were analysed using light microscopy to determine the egg counts for S. mansoni and STH. The numbers of eggs per slide were used to obtain a measure of the number of helminth eggs per gram of faeces (EPG).*

31. All studies announced are not referenced

***Response****: All primary studies included in this systematic review were appropriately referenced in Table 1, using the superscript numerals in parentheses.*

32. Two sub title overlap. line 334 Anaemia in Plasmodium- helminth co-infections" and line 365: Anaemia induced by malaria-helminth co-infection"

***Response****: We have revised the two sub-titles as highlighted above*

33. Other reviews on co-infection should be reviewed to avoid the redundant since some facts are known already.

***Response****: We have considered carefully other existing systematic reviews on malaria-helminth co-infection and avoided duplication of facts that are already known about the subject*
